# The Association Between Venous Invasion and Distant Metastasis in Head and Neck Squamous Cell Carcinoma

**DOI:** 10.7759/cureus.78023

**Published:** 2025-01-26

**Authors:** Takumi Okuda, Takayuki Kawabata

**Affiliations:** 1 Otolaryngology-Head and Neck Surgery, Miyazaki Prefectural Miyazaki Hospital, Miyazaki, JPN

**Keywords:** distant metastasis, head and neck squamous cell carcinoma, lymphatic invasion, microvascular invasion, venous invasion

## Abstract

Background: In surgically treated cases of head and neck squamous cell carcinoma (HNSCC), even pathological N0 (pN0) cases according to the Tumor, Node, and Metastasis (TNM) Classification, distant metastases can occur relatively early postoperatively. Therefore, we hypothesized that hematogenous distant metastasis may be related to the degree of venous invasion. Lymphatic invasion is considered to be a poor prognostic factor in HNSCC, but knowledge about venous invasion is scarce. We therefore investigated the association between venous invasion and distant metastasis, an important poor prognostic factor, in cases of HNSCC from our institution.

Subjects and methods: Between April 2020 and December 2023 (four years and nine months), there were 89 cases in which a postoperative pathological evaluation of microvascular invasion was conducted after HNSCC surgery at our institution. Of these, 73 were retrospectively reviewed, after excluding six cases with positive margins and 10 cases with extranodal extension of metastatic lymph nodes. The observation period ranged from 13 to 54 months (mean: 32.5 months). The correlations between the presence/absence of venous or lymphatic invasion at the primary site and distant metastasis were investigated.

Results: Among the 73 cases, venous invasion was found at 31 primary sites, and lymphatic invasion was observed in 38 cases. Distant metastases were found in 10 cases. All cases showed both venous out lymphatic invasion, and none of the cases where these were negative showed distant metastasis. There were significantly more distant metastases in both the venous invasion-positive group (p=0.001) and the lymphatic invasion-positive group (p=0.004).

Conclusion: The absence of distant metastasis in cases that were negative for venous invasion and venous invasion being present in all cases with distant metastasis indicated that venous invasion by the primary tumor is an important factor in distant metastasis. Venous invasion was also found to increase in frequency as the T and N stages progressed.

## Introduction

In surgically treated cases of head and neck squamous cell carcinoma (HNSCC), even pathological N0 (pN0) cases according to the Tumor, Node, and Metastasis (TNM) Classification, distant metastases occur relatively early postoperatively. Therefore, we hypothesized that hematogenous distant metastasis may be related to the degree of venous invasion by the primary tumor. In Japan, the histological findings of tissue excised from HNSCC are reported according to the General Rules for Clinical Studies on Head and Neck Cancer (6th edition, revised version) (Japanese), which require information about the resection margins (pathological horizontal margin (pHM), pathological vertical margin (pVM)), vascular invasion (venous invasion (v), lymphatic invasion (ly)), and perineural invasion (pn) to be reported. The degree of vascular invasion is classified into four levels from 0 to 3 (lymphatic invasion: ly0-3, venous invasion: v0-3). Lymphatic invasion in HNSCC is already considered to be a poor prognostic factor [1-3], but knowledge about venous invasion is scarce. We therefore investigated the association between venous invasion and distant metastasis, an important poor prognostic factor, in cases of HNSCC from our institution.

## Materials and methods

This study was conducted at Miyazaki Prefectural Miyazaki Hospital in Miyazaki, Japan, after obtaining approval from the institution's Ethical Review Board (approval number: 24-46).

Between April 2020 and December 2023 (four years and nine months), there were 89 cases in which a postoperative pathological evaluation of microvascular invasion was conducted after HNSCC surgery at our institution. Of these, 73 were retrospectively reviewed, after excluding six cases with positive margins and 10 cases with extranodal extension of metastatic lymph nodes. The present study was not limited to N0 cases. This was because there were clinical N0 cases in which only the primary tumor was resected or prophylactic dissection was performed, and some of these latter cases were pathological N-positive. Sixty-five patients were male, and eight were female, and their ages ranged from 34 to 91 years (mean: 70.6 years). The duration of the observation period ranged from 13 to 54 months (mean: 32.5 months). It was defined as the period from the date of surgery to the date of the last medical examination. The presence or absence of venous invasion was determined by Elastica-van-Gieson staining, using purple-black-stained elastic fibers as an indicator. The presence/absence of lymphatic invasion was determined by D2-40 staining. The correlations between the presence/absence of venous or lymphatic invasion at the primary site and distant metastasis were investigated. The correlations between the presence/absence of vascular invasion (assessed separately for lymphatic and venous invasion) and T and N classification stages were also examined. The T and N classifications were based on pathological findings as a rule, while the N classification was the clinical N classification in cases in which only the primary site was resected. The degree of vascular invasion was classified into four levels (lymphatic invasion: ly0-3, venous invasion: v0-3) from 0 to 3 according to the General Rules for Clinical Studies on Head and Neck Cancer (6th edition, revised version) (Table 1), but in this study, the invasion-negative (0) and invasion-positive (1-3) groups were compared.

**Table 1:** Description of histological findings based on the General Rules for Clinical Studies on Head and Neck Cancer (6th edition, revised version) Note: Very mild refers to, for example, searching all prepared sections and finding only some of the targeted findings (one or two lesions in all specimens), while extremely high degree refers to many sections having the targeted findings (findings in almost all prepared sections).

Description of histological findings	
Lymphatic invasion	
ly0	No lymphatic invasion
ly1	Very mild lymphatic invasion
ly2	An intermediate degree of lymphatic invasion (between ly1 and ly3)
ly3	An extremely high degree of lymphatic invasion
Venous invasion	
v0	No venous invasion
v1	Very mild venous invasion
v2	An intermediate degree of venous invasion (between v1 and v3)
v3	An extremely high degree of venous invasion
Perineural invasion	
pn0	No evidence of perineural invasion
pn1	Very mild perineural invasion
pn2	An intermediate degree of perineural invasion (between pn1 and pn3)
pn3	An extremely high degree of perineural invasion

Statistical analyses were performed with IBM SPSS Statistics for Windows, Version 26.0 (Released 2019; IBM Corp., Armonk, New York, United States) using Student's t-test (age, observation period), Wilcoxon's signed rank sum test (T classification, N classification), and the chi-squared test (distant metastasis, sex). A p-value of less than 0.05 was considered statistically significant.

## Results

Among the 73 cases for which follow-up data were available (oral cavity (35), maxillary sinus (one), oropharynx (nine), hypopharynx (16), larynx (11), and external auditory canal (one)), 31 primary sites were found to have venous invasion (v1-3), and 42 had no evidence of venous invasion (v0) (Table 2).

**Table 2:** Association between venous invasion and distant metastasis *A p-value of less than 0.05 was considered statistically significant.

	v0 (n=42)	v1-3 (n=31)	P-value
Distant metastasis	0	10	<0.05*
Mean age (years)	69.3 (34-91)	72.4 (50-88)	0.16
Male-to-female ratio	37:5	28:3	0.76
Mean pathologic T stage	1.9	2.9	<0.05*
Mean pathologic N stage	0.6	1.1	<0.05*
Average follow-up (months)	35.4 (17-54)	28.5 (13-53)	0.02

Lymphatic invasion was observed in 38 cases (ly1-3), but not in 35 cases (ly0) (Table 3).

**Table 3:** Association between lymphatic invasion and distant metastasis *A p-value of less than 0.05 was considered statistically significant.

	ly0 (n=35)	ly1-3 (n=38)	P-value
Distant metastasis	0	10	<0.05*
Mean age (years)	70.2 (34-91)	71.0 (50-88)	0.78
Male-to-female ratio	31:4	34:4	0.9
Mean pathologic T stage	1.9	2.7	<0.05*
Mean pathologic N stage	0.3	1.2	<0.05*
Average follow-up (months)	33.5 (15-54)	31.5 (13-54)	0.51

Distant metastases were found in 10 cases (nine in the lungs, four in the liver, and one in the bone; some cases involved multiple sites) (Table 4).

**Table 4:** Characteristics of distant metastasis cases ly: lymphatic invasion; v: venous invasion; EvG: Elastica-van-Gieson staining; pn: perineural invasion; pHM: pathological horizontal margin; pVM: pathological vertical margin; TL: total laryngectomy; Bil. ND: bilateral neck dissection; TPL: total pharyngolaryngectomy; TOVS: transoral videolaryngoscopic surgery; Rt.: right-sided; TTD: time to the detection of distant metastasis (postoperative months) *Of the 73 cases, there were no cases of distant metastasis in ly0 or v0 cases. **The average was 11.1 months.

Age	Sex	Primary site	Surgical method	pT	pN	pStage	*ly (D2-40)	*v (EvG)	pn	pHM	pVM	Distant metastasis	**TTD
65	M	Larynx	TL+Bil. ND	4a	2c	ⅣA	2	2	1	0	0	Lung, liver, bone	2
84	M	Larynx	TL+Bil. ND	4a	2c	ⅣA	3	2	2	0	0	Liver	3
61	M	Hypopharynx	TPL+Bil. ND	2	2c	ⅣA	1	2	2	0	0	Lung	4
50	M	Hypopharynx	TPL+Bil. ND	4a	2c	ⅣA	2	2	3	0	0	Lung	6
73	M	Larynx	TL+Bil. ND	3	0	Ⅲ	1	1	0	0	0	Lung, liver	8
61	M	Hypopharynx	TPL+Bil. ND	4a	2b	ⅣA	3	2	0	0	0	Lung	12
68	M	Larynx	TL+Bil. ND	4a	0	ⅣA	3	1	0	0	0	Lung, liver	17
73	M	Oropharynx (p16-)	TOVS+Rt. ND	2	2b	ⅣA	1	1	0	0	0	Lung	19
73	M	Oral	Hemiglossectomy+Rt. ND	3	2b	ⅣA	1	1	0	0	0	Lung	20
67	M	Hypopharynx	TPL+Bil. ND	3	2b	ⅣA	1	1	3	0	0	Lung	20

All cases showed both venous out lymphatic invasion, and none of the cases where these were negative showed distant metastasis (Table 5). There were two pN0 cases among the cases in which distant metastases developed. The time at which distant metastases were detected ranged from two to 20 postoperative months (mean: 11.1 postoperative months).

**Table 5:** Venous and lymphatic invasion and distant metastasis

	ly0 (n=35)	ly1-3 (n=38)	Distant metastasis
v0 (n=42)	27	15	0
v1-3 (n=31)	8	23	10
Distant metastasis	0	10	Total 10

For venous invasion, the duration of the observation period was significantly longer in the negative group (p=0.02), and there were significantly (p=0.001) more distant metastases in the positive group. There were no significant differences in the age or sex distribution between the two groups. There were no significant differences in age or sex distribution or the observation period between the two lymphatic invasion groups, although there were significantly (p=0.004) more distant metastases in the positive group. Regarding the TN classification, the stage was significantly higher in the positive group for both venous and lymphatic invasion.

## Discussion

In terms of the pathological assessment of tumor tissue and its clinical relevance, lymphatic invasion is generally associated with lymph node metastasis, venous invasion is associated with distant metastasis, and perineural invasion is associated with local recurrence. Lymphatic invasion has been widely reported in HNSCC and has already been shown to be a poor prognostic factor [1-3].

On the other hand, venous invasion in HNSCC has not been assessed in detail based on routine histological examinations, and its prognostic significance is unknown. This is due to the difficulty of assessing venous invasion with hematoxylin and eosin staining; however, in recent years, the widespread use of special stains that target the elastic fibers found in vein walls, such as Elastica-van-Gieson stain and Victoria Blue, has made it possible to assess venous invasion with high accuracy [4]. Using this approach, Gopinath et al. recently reported increased local recurrence and decreased recurrence-free survival in cases of HNSCC in which the primary tumor exhibited venous invasion [5]. They examined 105 lymph node-negative cases (pathological and clinical N0) to avoid the prognostic impact of lymph node metastases. However, we included lymph node-positive cases because, in addition to the reasons given in the subjects and methods section, we consider that lymph node metastasis is also associated with distant metastasis due to venous invasion, as discussed later. However, cases that exhibited positive extranodal extension were excluded from this study in order to prevent them from having a strong influence on distant metastasis, as it was considered highly likely that residual tumor tissue would remain after surgery in such cases, as is true in cases with positive primary tumor resection margins. Previous reports have also reported a significantly worse prognosis in cases of positive extranodal extension of metastatic lymph nodes [6-8].

In gastric cancer, venous invasion is considered to signal the beginning of hematogenous metastasis, and the Japanese classification of gastric carcinoma requires clinicians to search for it [9]. Venous invasion has also been established as an independent prognostic indicator of hematogenous metastasis and reduced survival in colorectal cancer [10,11]. It is similarly considered to be a prognostic indicator in breast cancer [12,13]. On the other hand, in a study of adenocarcinoma or squamous cell carcinoma of the lung, the presence of venous invasion was not associated with survival, while lymphatic invasion was associated with a poor prognosis [14]. In a study of rectal neuroendocrine tumors, there were few cases of metastasis or recurrence that resulted in death among vascular invasion-positive cases that were diagnosed using immunohistochemical staining or special stains, and the clinical impact of vascular invasion by rectal neuroendocrine tumors is unknown [15-17]. The relationships between venous invasion by the primary tumor and distant metastasis and prognosis currently appear to vary from organ to organ and even carcinoma to carcinoma within the same organ.

Therefore, we assessed the association between venous invasion and distant metastasis in HNSCC, as there is no doubt that distant metastasis is an important prognostic factor. To the best of our knowledge, this is the first study to assess the association between venous invasion and distant metastasis in HNSCC. In the present study, cases that were negative for venous invasion did not show distant metastasis, while all of the cases with distant metastasis were positive for venous invasion. Surprisingly, the same was also true for lymphatic invasion. Therefore, it is not known which is the more important factor for distant metastasis or whether both are important. However, both types of invasion were shown to be important factors for distant metastasis in HNSCC. The frequency of both venous and lymphatic invasion was found to increase as the T and N stages progressed. We suspect that venous invasion is important for distant metastasis and that as the N stage progresses, venous invasion within metastatic lymph nodes also increases, which may be the cause of the distant metastasis seen in lymphatic invasion-positive cases. This will become clearer in the future when the pathology of venous invasion in metastatic lymph nodes is assessed. In Japan, the degree of vascular invasion is classified into four levels from 0 to 3 (Table 1), but in this study, due to sample size limitations, comparisons were made between the invasion-negative (0) and invasion-positive (1-3) groups. In the future, we would like to continue studying our case series and also consider the risk of distant metastasis according to the degree of invasion positivity.

With the above points in mind, clinicians should consider when to perform imaging studies in the postoperative follow-up period and the suitability of further treatment.

The limitations of this study included the fact that it was a small, single-center, retrospective study and the short observation period. The observation period in this study ranged from 13 to 54 months (mean: 32.5 months), and as 80% of recurrent metastatic cases of HNSCC generally occur within 12-24 months, the present results may be reasonable. However, to obtain more definitive results, multicenter, prospective studies involving larger cohorts with a sufficient observation period are desirable.

## Conclusions

Based on 73 cases of HNSCC treated at our institution, the association between venous invasion by the primary tumor and distant metastasis, an important poor prognostic factor, was retrospectively examined. The absence of distant metastasis in cases that were negative for venous invasion and the presence of venous invasion in all cases with distant metastasis indicated that venous invasion by the primary tumor is an important factor in distant metastasis. Venous invasion was also found to increase in frequency as the T and N stages progressed.
